# Quality of life is predictive of relapse in schizophrenia

**DOI:** 10.1186/1471-244X-13-15

**Published:** 2013-01-09

**Authors:** Laurent Boyer, Aurelie Millier, Emeline Perthame, Samuel Aballea, Pascal Auquier, Mondher Toumi

**Affiliations:** 1 Aix-Marseille Univ, EA 3279 Research Unit, Marseille, 13284, France; 2 Creativ-Ceutical France, rue du Faubourg Saint-Honoré, Paris, 75008, France; 3 Decision Sciences & Health Policy, Boulevard du 11 Novembre 1918, UCBL 1 - Chair of Market Access University Claude Bernard Lyon I, Villeurbanne, 69622, France

**Keywords:** Schizophrenia, Quality of life, Relapse, Compliance, Functioning, Recovery

## Abstract

**Background:**

The objective of this study was to evaluate whether quality of life (QoL), as measured by the SF36 and the Quality of Life Interview (QoLI), is predictive of relapse for patients with schizophrenia.

**Methods:**

Using data from a multicenter cohort study conducted in France, Germany, and the United-Kingdom (EuroSC), we performed Cox proportional-hazards models to estimate the associations between QoL at baseline and the occurrence of relapse over a 24-month period, with adjustment for age; gender; positive, negative and general psychopathology PANSS factors; functioning (GAF); medication; side-effects; and compliance measures.

**Results:**

Our sample consisted of 1,024 patients; 540 (53%) had at least one period of relapse, and 484 (47%) had no relapse. QoL levels were the most important features predicting relapse. We found that a higher level of QoL predicts a lower rate of relapse at 24 months: HR = 0.82 (0.74; 0.91), p < 0.001 for the SF36-Physical Composite Score; and HR = 0.88 (0.81; 0.96), p = 0.002 for the SF36-Mental Composite Score. These results were not confirmed using the QoLI: HR = 0.91 (0.81; 1.01), p = 0.083. To a lesser extent, older age, better functioning, and a higher compliance score also predict a lower rate of relapse at 24 months (HRs from 0.97 to 0.98; p < 0.05).

**Conclusions:**

QoL, as assessed by the SF36, is an independent predictor of relapse at a 24-month follow-up in schizophrenia. This finding may have implications for future use of the QoL in psychiatry. Moreover, our findings may support the development and monitoring of complementary therapeutic approaches, such as ‘recovery-oriented’ combined with traditional mental health cares to prevent relapse.

## Background

Schizophrenia is a severe and chronic mental illness that is characterized by recurrent relapses [1,2]. Relapse is disabling and distressing for the individual with schizophrenia and is associated with a progressive functional deterioration as well as worsening treatment response and clinical prognosis [3]. Moreover, relapse increases caregiver burden [4] and represents a significant economic burden on families and society [5,6]. Because the prevention of relapse is a major challenge in the care of patients with schizophrenia, numerous studies have investigated the value of socio-demographic, clinical, and medication factors in the prediction of relapse [1,2,5,7-9]. However, although these reports have provided a better understanding of factors that influence the course of schizophrenia, these factors imperfectly predict relapse, and the relapse rate still remains high. Other factors, such as social and environmental factors known to influence the course of schizophrenia [10,11], have received scant attention. A more thorough and comprehensive understanding of these factors is necessary. On the other hand, Quality of Life (QoL), which is defined as a subjective evaluation embedded in a cultural, social, and environmental context [12], has gained increasing acceptance in psychiatric research along with the traditional assessments of clinical outcomes. Recent studies have shown QoL to be an independent prognostic factor associated with clinical outcome in various chronic diseases including oncology, often predicting survival or occurrence of hospitalization [13-17]. However, two studies have shown that QoL scores added relatively little to socio-demographic, clinical, and medication factors in the prediction of relapse in schizophrenia [1,5]. Because the median time to relapse following a previous remission in schizophrenia has been estimated to occur at the end of the first year [18,19], the relatively short follow-up period of these two studies (6 and 12 months) may have underestimated the rate of relapse. To date, no study has assessed whether QoL provides prognostic information in addition to conventional socio-demographic, clinical, and medication factors for patients with schizophrenia after a sufficient follow-up. The objective of this study was to evaluate whether baseline QoL, as measured by the SF36 and the QoLI, is predictive of relapse for patients with schizophrenia over a 24-month follow-up.

## Methods

### Study design and sampling

The data are from the European Schizophrenia Cohort (EuroSC) conducted in France, Germany, and the United Kingdom (UK). A detailed description of the EuroSC has been published previously [20]. It is a naturalistic 2-year follow-up, from 1998 to 2000, of a cohort of patients suffering from schizophrenia. The study was observational, as no intervention was made either by or at the behest of the research team. The main objective of the EuroSC was to identify and describe the types of treatment and methods of care for individuals with schizophrenia and to correlate these with clinical outcomes, states of health, and quality of life. This study was conducted in accordance with the Declaration of Helsinki and the French Good Clinical Practices. The protocol of this study was approved by the Institutional Review Board or the Ethics Committee responsible for the participating hospital or institution: The Amden & Islington Community Mental Health NHS Trust Ethics Committee and The Leicester University Committee for Research Ethics for all UK sites, The Ethics Committee of the University of Leipzig for Germany, and The Ethics Committee of the University of Aix-Marseille 2 for France. Written informed consent was obtained from each participant after the study details had been fully explained.

In France, participants were recruited from 4 areas: Lille (Northern France), Lyon, Clermont-Ferrand (Central France), Marseille and Toulon (Southern France). In Germany, the study took place in 4 areas: Leipzig and Altenburg in the former East Germany, and the districts of Hemer and Heilbronn in the former West Germany. In the UK, the two centers of Islington, an inner-city area of London, and the county of Leicestershire (excluding the city of Leicester) were chosen. In each center, patients were identified according to the following criteria: diagnosis of schizophrenia according to the DSM-IV criteria [21], ages 18 to 64 years, and absence of relapse for the previous 12 months. Random sampling from these patients was used to generate a representative sample. The sample included 1,208 patients: 287 from France, 619 from Germany, and 302 from the UK. Five interviews were completed with each participant at initial assessment and after every 6 months for the subsequent 2 years. Sample attrition resulted in 1024 (84.8%) participants taking part in the second interview, 914 (75.7%) in the third, 777 (64.3%) in the fourth, and 684 (56.7%) in the final interview.

### Data collection

The following data were collected at baseline:

1. Socio-demographic information: gender, age, living conditions, and employment status.

2. Clinical characteristics: psychotic symptoms based on the Positive and Negative Syndrome Scale (PANSS), which includes three different subscales (positive, negative and general psychopathology) [22]; and functioning based on the Global Assessment of Functioning (GAF) scale [23].

3. Drug information: Antipsychotic medication (first-generation antipsychotics - FGAs, second-generation antipsychotics – SGAs); the Simpson and Angus Scale (SAS) [24], the Barnes Akathisia Scale (BAS) [25], and the Abnormal Involuntary Movement Scale (AIMS) [26] were used to assess side-effects; the Rating of Medication Influences (ROMI) Scale was used to evaluate adherence to treatment [27]. We only examined the responses to Part I of the ROMI, which assesses the reasons for taking the medication. In our analysis, however, we did not include Part 2 of the ROMI, which assesses the reasons why people might not take their medication. According to Weiden et al. [27], the non-compliance items in the ROMI apply only to patients who have not taken their medication for at least one week for any part of the past month; otherwise, only the compliance items are administered. The latter situation applied to all participants in this study.

4. QoL was assessed using 2 types of questionnaires: a generic measure usable regardless the health status of the individual (either healthy or with different health conditions) - the SF36 [28]; and a measurement specific to people with chronic mental illnesses, tailored to a broad range of mental illnesses - the Quality of Life Interview (QoLI) [29].

The SF36 is a generic, self-administered QoL questionnaire consisting of 36 items describing 8 dimensions: Physical Functioning (PF); Social Functioning (SF); Role—Physical Problems (RPP); Role—Emotional Problems (REP); Mental Health (MH); Vitality (VIT); Bodily Pain (BP); and General Health (GH). Two composite scores can be calculated: the physical composite score (SF36-PCS) and the mental composite score (SF36-MCS). Each dimension is scored within a range from 0 (low QoL level) to 100 (high QoL level).

The QoLI is an instrument specifically designed to assess QoL in patients with severe mental illnesses. It consists of 74 items describing 8 domains: Living Situation (LS), Daily Activities and functioning (DA), Family Relationships (FR), Social Relationships (SR), Finances (F), Work and School (WS), Legal and Safety Issues (LSI), and Mental and Physical Health (MPH). Each domain is rated objectively by an interviewer and subjectively by the patient reporting his/her satisfaction through an individual structured interview. Subscale scores and an overall life satisfaction (OLS) score are calculated, ranging from 1 to 7. Higher scores indicate a better QoL.

For the purpose of the study, only the SF36-PCS, SF36-MCS, and QoLI-OLS were included in the analyses.

### Study outcomes

Our primary measure was the time to first relapse during a 24-month period. Relapse was defined according to a common, clinically reproducible and validated definition [30,31]: (1) hospitalization due to worsening of psychotic symptoms or an unequivocal worsening of psychotic symptoms of such magnitude that hospitalization appeared imminent, or (2) a re-emergence of florid psychotic symptoms such as delusions, hallucinations, or bizarre behavior, or (3) a thought disorder lasting seven days or more. This information was obtained by a structured clinical interview, centred on a checklist of criteria, conducted by a psychiatrist every six months. Relapse was defined relative to the baseline characteristics of the patient. Additional relevant information was obtained from medical records and through staff interviews. This process was intended to standardise the collection of information about relapse.

### Statistical analysis

Descriptive statistics, reported as means (SD) or percentages, summarized baseline characteristics by relapse status. Characteristics of patients were compared using Chi-squared or Fisher exact tests for categorical variables and the Student or Wilcoxon rank sum test for continuous variables. Cox proportional hazards models were also used for the univariate analyses to predict the interval time to relapse. Subjects were censored from the survival analysis at the time they discontinued the study, i.e. at the time of last interview.

Multivariate Cox proportional-hazards models were performed to estimate the Hazard Ratio (HR) and its corresponding 95% confidence interval (CI) for associations between QoL scores and the occurrence of relapse, with adjustment for baseline characteristics, one model including the SF36 questionnaire (SF36 model), and one model including the QoLI (QoLI model). The adjustment variables relevant to the models were selected from the univariate analysis, based on a threshold p-value ≤0.20 (age, positive, negative and general psychopathology PANSS scores, GAF score, medication, side-effects based on the BAS score, compliance ROMI score). An additional variable was included in the models owing to its socio-demographic interest (gender). The proportional hazards assumption was investigated by testing the constancy of the log hazard ratio over time by means of log-minus-log survival plots; according to the test, the proportional hazard assumption was not violated. The statistical significance level was set at p < 0.05 in a two-sided test. The SAS statistical package (SAS System for Windows, version 9.1) was used to perform all analyses.

## Results

Of the 1,208 patients in the EuroSC, 184 patients were excluded from the analysis because they did not have any follow-up data (15%). A total of 1,024 patients (85%) were then included in the present analysis. There were no statistical differences between the 1,024 patients in the study group and those 184 who were excluded in terms of age, gender, living condition, functioning (GAF score), antipsychotic medication, SAS, BAS AIMS, and QoL scores at baseline (all p-values > 0.05). On the contrary, in comparison to included patients, excluded patients had a slightly higher severity of symptoms (respectively 56.5 (20.2) vs. 64.3 (24.9), p = 0.001 for the total PANSS score) and lower compliance scores (respectively 11.0 (3.3) vs. 11.5 (3.1), p = 0.043 for the ROMI compliance score).

### Baseline characteristics of patients with relapse and no relapse

The baseline characteristics of the patients with relapse and no relapse are reported in Table 1. Five hundred and forty patients (53%) had at least one period of relapse, and 484 (47%) had no relapse. Patients with relapse were significantly younger than patients with no relapse, but no significant difference was found for gender and living conditions. Patients with relapse also had higher levels of severity (positive, negative, and general psychopathology PANSS factors) and a lower level of functioning. The proportion of patients receiving SGAs was significantly higher in patients with relapse than in patients with no relapse. Side effects as assessed with the AIMS, BAS, and SAS and compliance as assessed with the ROMI did not differ significantly between the two groups. Concerning QoL scores, patients with relapse reported lower QoL levels than patients with no relapse for the SF36-PCS, SF36-MCS, and QoLI-OLS.

**Table 1:** Characteristics of the patients according to relapse status and univariate Cox proportional-hazards models: Crude Hazard Ratio (HR) and its corresponding 95% confidence interval (95% CI)

	***Relapse***					
	**Yes (N = 540)**	**No (N = 484)**				
	**M (SD)**^**1**^	**M (SD)**	**p-value**	**Crude HR**	**95% CI**	**p-value**
Socio-demographic characteristics						
Age	40.3 (10.1)	42. 0 (11.6)	p = 0.013	0.99	(0.98;0.99)	p = 0.004
Gender (Male), N (%)^2^	62.1	61.2	p = 0.773	0.97	(0.82;1.16)	p = 0.762
Living alone, N (%)	33.5	34.5	p = 0.740	0.97	(0.81;1.16)	p = 0.754
Unemployed, N (%)	56.9	35.7	P = 0.240	0.79	(0.53;1.15)	p = 0.237
Severity of symptoms: PANSS^3^						
Positive PANSS score	12.7 (5.7)	11.7 (5.1)	p = 0.003	1.03	(1.01;1.04)	p = 0.001
Negative PANSS score	16.1 (7.3)	14.6 (7.5)	p = 0.002	1.02	(1.01;1.03)	p = 0.001
General Psychopathology PANSS score	29.7 (10.2)	27.7 (10.1)	P = 0.001	1.02	(1.01;1.02)	p < 0.001
Functioning						
GAF^4^	48.5 (15.6)	55.0 (15.9)	p < 0.001	0.98	(0.98;0.99)	p < 0.001
Side Effects						
BAS^5^	1.3 (3.0)	1.1 (2.7)	p = 0.192	1.02	(0.99;1.04)	p = 0.147
AIMS^6^	3.0 (6.9)	2.5 (5.6)	p = 0.228	1.01	(0.99;1.02)	p = 0.328
SAS^7^	3.5 (8.2)	3.5 (7.7)	p = 0.963	1.00	(0.99;1.011)	p = 0.990
Medication						
Second-generation antipsychotics (Yes), N (%)	45.6	38.9	p = 0.034	1.26	(1.06;1.49)	p = 0.009
Attitude towards medication: ROMI^8^						
ROMI compliance score	10.8 (3.5)	11.1 (3.1)	p = 0.223	0.97	(0.95;0.99)	p = 0.035
Quality of life						
SF36 - Physical Composite Score *	47.2 (9.8)	49.1 (8.7)	p = 0.001	0.83	(0.76;0.91)	p < 0.001
SF36 - Mental Composite Score **	40.3 (11.8)	43.7 (11.0)	p < 0.001	0.84	(0.78;0.91)	p < 0.001
QoLI - OLS score***	4.7 (0.9)	4.8 (0.8)	p = 0.006	0.86	(0.78;0.95)	p = 0.003

^1^ Mean (Standard Deviation); ^2^ Effective (Percentage); ^3^ Positive and Negative Syndrome Scale; ^4^Global Assessment Functioning; ^5^Barnes Akathisia score; ^6^Abnormal Involuntary Movement Score; ^7^Simpson-Angus score; ^8^Rating of Medication Influences Scale.

* HR for the SF36 - Physical Composite Score should be interpreted this way, “an increase of 10 points on the Physical Composite Score multiplied by 0.83 the instantaneous risk of relapse at 24 months”;

** HR for the SF36 - Mental Composite Score should be interpreted this way, “an increase of 10 points on the Mental Composite Score multiplied by 0.84 the instantaneous risk of relapse at 24 months”;

*** HR for the QoLI - OLS score should be interpreted this way, “an increase of 1 point on the QoLI score multiplied by 0.86 the instantaneous risk of relapse at 24 months”.

### Predictors of relapse in the Cox’s proportional hazard models

In the univariate Cox’s proportional hazard models analysis (Table 1), relapse was significantly predicted by older age, higher level of severity, lower level of functioning, SGAs, lower compliance and lower QoL level.

Factors that were independently associated with relapse during the 24 months of follow-up on the basis of the multivariate Cox analyses are reported in Table 2. In the SF36 model, QoL levels were the most important features predicting relapse. A higher level of QoL predicts a lower rate of relapse at 24 months for both the SF36-PCS and -MCS. To a lesser extent, older age, better functioning, and higher compliance score also predict a lower rate of relapse at 24 months. On the contrary, the severity of symptoms and second-generation antipsychotics did not significantly predict relapse. In the QoLI model, only older age and better functioning predict a lower rate of relapse.

## Discussion

Using data from the observational EuroSC cohort, we examined the predictive value of QoL with respect to relapse. Our study examined 1,024 patients with schizophrenia; controlled for important socio-demographic, clinical, and medication factors; and has attempted to overcome the limitations of past studies by using a large sample size and a 24-month follow-up, which enables a more complete analysis of full relapse. The findings provide evidence in support of a relationship between QoL scores and relapse in this patient population.

**Table 2:** Factors associated with relapse during the 24 months of follow-up in the multivariate Cox proportional-hazards models: Adjusted Hazard Ratio (HR) and its corresponding 95% confidence interval (95% CI)

	**SF-36 Model**			**QoLI Model**		
	**HR**	**95% IC**	**p-value**	**HR**	**95% IC**	**p-value**
Quality of life						
SF36 - Physical Composite Score *	0.82	(0.74;0.91)	p < 0.001	-	-	-
SF36 - Mental Composite Score **	0.88	(0.81;0.96)	p = 0.002	-	-	-
QoLI - OLS score***	-	-	-	0.91	(0.81;1.01)	p = 0.083
Socio-demographic characteristics						
Age	0.98	(0.98;0.99)	p = 0.001	0.98	(0.98;0.99)	p < 0.001
Gender (Male)	1.06	(0.88;1.30)	p = 0.532	1.12	(0.93;1.34)	p = 0.249
Severity of symptoms: PANSS^1^						
Positive PANSS score	0.99	(0.97;1.02)	p = 0.514	0.99	(0.97;1.02)	p = 0.560
Negative PANSS score	1.01	(0.99;1.03)	p = 0.248	1.00	(0.98;1.02)	p = 0.969
General Psychopathology PANSS score	0.99	(0.97;1.01)	p = 0.419	1.00	(0.98;1.02)	p = 0.937
Functioning						
GAF^2^	0.98	(0.97;0.99)	p < 0.001	0.98	(0.97;0.99)	p < 0.001
Side Effects						
BAS^3^	0.99	(0.95;1.02)	p = 0.497	1.00	(0.97;1.03)	p = 0.844
Medication						
Second-generation antipsychotics (Yes)	1.07	(0.88;1.29)	p = 0.508	1.13	(0.94;1.36)	p = 0.195
Attitude towards medication: ROMI^4^						
ROMI compliance score	0.97	(0.94;0.99)	p = 0.041	0.97	(0.95;1.00)	p = 0.063

^1^ Positive and Negative Syndrome Scale; ^2^Global Assessment Functioning; ^5^Barnes Akathisia score; ^4^Rating of Medication Influences Scale.

* HR for the SF36 - Physical Composite Score should be interpreted this way, “an increase of 10 points on the Physical Composite Score multiplied by 0.82 the instantaneous risk of relapse at 24 months”;

** HR for the SF36 - Mental Composite Score should be interpreted this way, “an increase of 10 points on the Mental Composite Score multiplied by 0.88 the instantaneous risk of relapse at 24 months”;

*** HR for the QoLI - OLS score should be interpreted this way, “an increase of 1 point on the QoLI score multiplied by 0.91 the instantaneous risk of relapse at 24 months”.

QoL, as assessed by a generic instrument (SF36), is an independent predictor of relapse in schizophrenia. This finding is consistent with a 12-month follow-up study using the SF36, in which a higher level of QoL predicted a moderately lower rate of relapse at 12 months (OR = 0.98 for both the Physical and Mental Composite Scores) [1]. Surprisingly, this relationship was not confirmed in our study using the QoLI, which only presented a trend (p = 0.083). Again, this finding is consistent with a 6-month follow-up study using the QoLi, in which relapsed patients appeared to experience a lower QoL than non-relapsed patients at baseline, but the differences were not statistically significant [5]. The fact that a generic instrument like the SF36 better predicts relapse than a specific instrument like QoLI may appear paradoxical. The use of QoL-specific instruments is generally recommended in schizophrenia because they identify the specific needs of patients and are more sensitive to change and treatment/intervention effects than are generic instruments [32]. However, the QoLI is designed to encompass a broad range of mental illnesses, not specifically schizophrenia. According to Cramer et al. [32], the QoLI showed less sensitivity to change and treatment effect than did schizophrenia specific QoL instruments. A previous study has also shown that a better agreement was observed between the SF36 and schizophrenia specific QoL instruments than with the QoLI and that the SF-36 was more strongly correlated with clinical status than the QoLI [33].

An important finding in our study concerns the SF36-PCS, which was a stronger relapse predictor compared to the SF36-MCS. This finding confirms the need for clinicians to increase their attention on the physical health of patients with schizophrenia [34]. Although most patients view their physical health as a high priority, many clinicians consider their primary function to be the management of mental and psychological health [35]. The subjective physical well-being of patients with schizophrenia should thus be considered by clinicians as an important predictor of relapse, in the same way that psychological aspects are considered.

In our study, unlike previous studies [5,7,19,36], a more severe symptomatology was not associated with relapse, and other factors such as functioning or compliance were only moderately associated with relapse. One possible explanation for this discrepancy might be that QoL, which was not taken into account as a potential predictor in previous studies, may have a confounding influence on the relationship between traditional predictors and relapse. On the other hand, the exclusion of more severe and less compliant patients (15% of the cohort) may have also attenuated the predictor strength of symptomatology and compliance. Beyond these explanations, it is interesting to note that, although the predictive value of the different factors was moderate in our study, QoL was a stronger predictor than clinical information.

Finally, our findings can also be interpreted in terms of the recovery process. The concept of recovery is broadly organised into 2 types: clinical objective (i.e. clinical symptoms and functioning) and personal subjective including in particular QoL among other domains such as personal confidence and hope, willingness to ask for help [37,38]. Our findings suggest that personal subjective recovery (i.e. QoL in our study) is more predictive of relapse than objective recovery. These findings should however be confirmed on other domains of subjective recovery. On the other hand, these results should be considered to improve the characterisation and treatment of patients with schizophrenia. Our findings may support the development and monitoring of complementary therapeutic approaches, such as ‘recovery-oriented’ combined with traditional mental health cares to prevent relapse. Interestingly, QoL has been linked to metacognitive capacities in recent studies [39,40], suggesting that interventions targeting metacognition, such as psychotherapy, may play a key role in preventing relapse [41].

### Limitations

Some limitations of this study have to be carefully considered.

First, a problem remains with the definition of relapse for schizophrenic patients. There are no consensual criteria for relapse [30]. Admission to a psychiatric hospital unit, increase in medication, worsening of florid symptoms of schizophrenia, worsening of any psychiatric symptoms, and threatened clinical exacerbations have all been variables used to indicate relapse [42]. However, in this study, we have chosen the most commonly used definition in the recent scientific literature.

Second, the representativeness of our sample should be discussed. Although the sampling procedure for the EuroSC aimed to provide a representative sample of the patients treated, this cohort of patients had mostly paranoid schizophrenia and was characterized by long-term illness [20]. Moreover, excluded patients presented with a higher clinical severity than included patients. Replication is therefore needed, using larger and more diverse groups of patients.

Third, 43% is a moderately high attrition rate in our study at 24 months, and this can lead to biased estimates, especially if patients who did not follow up failed to do so because they suffered a higher relapse rate due to higher clinical severity. However, three facts suggest minimal bias due to attrition. First, among patients lost to follow up, 40.3% had a relapse before their last visit and were thus included in the analysis. Second, patients lost to follow up did not differ significantly in age, gender, or PANSS scores at baseline from those patients who did follow up (p > 0.05; data not shown). Third, the relapse rate was relatively high (53%), suggesting that relapses were globally detected. Finally, attrition and relapse rates were globally within the range of other schizophrenia cohorts [1,5,6,43].

Fourth, the SF36 is a generic measure and may not adequately capture all areas of functioning and well-being that are relevant to people with schizophrenia [44]. Moreover, a recent review raised question about metrological properties of the SF36 in schizophrenia [45]. Future investigators should attempt to replicate our findings using disease-specific instruments.

Fifth, compliance is not easy to detect and quantify, and all methods of detection have some drawbacks. As such, the use of the ROMI may be criticised. This scale is a subjective method of assessing compliance in comparison with objective methods such as pill counts, pharmacy records, electronic monitor and plasma concentrations. However, as suggested by Velligan et al., even the use of more objective measures can result in significant errors [46]. Moreover, the ROMI has several advantages. It has good psychometric properties and predicts compliance satisfactorily [27].

Sixth, although our models account for a large set of potentially relevant variables, other factors might have increased their explanatory power. For example, having a history of previous relapse or hospitalisation was not a variable that we examined in our study, although it has previously been identified as an important predictor of relapse [1]. However, in the present study, patients did not have any relapse or hospitalisation for the 12 months prior to the baseline evaluation in accordance with the inclusion criteria. Lastly, despite our large sample, this study only included 3 European countries. Given the important influence of cultural, social, and environmental context on QoL, it would be necessary to know whether our findings can be replicated in other countries.

## Conclusion

This study shows that QoL measures can be considered as an independent predictor of relapse in schizophrenia. This finding may have implications for the future use of QoL in psychiatry. To date, QoL remains largely under-utilized in clinical practice [47]. Several studies have reported that clinicians believe that QoL measures lack clinical relevance for their patients [48,49]. More work must be done to convince clinicians of the clinical relevance of QoL instruments in order to enhance the use of QoL measures in clinical decision-making [50]. Our findings, similar to those in other studies, especially in oncology, provide strong support for the integration of QoL into clinical practice along with other standard assessments in psychiatry.

## Competing interests

The authors have declared that there are no conflicts of interest in relation to the subject of this study.

## Authors’ contributions

LB, AM, PA and MT wrote the manuscript. All authors designed the study and wrote the protocol. LB, AM, EP, and SA conducted the literature search and analyses. AM and EP conducted the statistical analyses. All authors contributed to and approved the final manuscript.

## Pre-publication history

The pre-publication history for this paper can be accessed here:

http://www.biomedcentral.com/1471-244X/13/15/prepub
